# Embedded Ethics in Practice: A Toolbox for Integrating the Analysis of Ethical and Social Issues into Healthcare AI Research

**DOI:** 10.1007/s11948-024-00523-y

**Published:** 2024-12-24

**Authors:** Theresa Willem, Marie-Christine Fritzsche, Bettina M. Zimmermann, Anna Sierawska, Svenja Breuer, Maximilian Braun, Anja K. Ruess, Marieke Bak, Franziska B. Schönweitz, Lukas J. Meier, Amelia Fiske, Daniel Tigard, Ruth Müller, Stuart McLennan, Alena Buyx

**Affiliations:** 1 Institute of History and Ethics in Medicine, Department of Preclinical Medicine, TUM School of Medicine and Health, Technical University of Munich, Ismaninger Straße 22, 81675 Munich, Germany; 2 Department of Science, Technology and Society (STS), School of Social Science and Technology, Technical University of Munich, Munich, Germany; 3 Institute of Philosophy & Multidisciplinary Center for Infectious Diseases, University of Bern, Bern, Switzerland; 4 TUD Dresden University of Technology, Dresden, Germany; 5 Department of Economics and Policy, School of Management, Technical University of Munich, Munich, Germany; 6 Amsterdam UMC, Department of Ethics, Law and Humanities, University of Amsterdam, Amsterdam, The Netherlands; 7 Churchill College, University of Cambridge, Cambridge, UK; 8 Department of Philosophy, University of San Diego, San Diego, USA; 9 Institute for Experiential AI, Northeastern University, Boston, USA; 10 Institute for Biomedical Ethics, University of Basel, Basel, Switzerland; 11 Center for Responsible AI Technologies, Technical University of Munich & University of Augsburg & Munich School of Philosophy, Munich, Germany; 12 Edmond & Lily Safra Center for Ethics, Harvard University, Cambridge, USA

**Keywords:** Ethics, Social science, Interdisciplinary collaborations, AI, Health, Technology

## Abstract

Integrating artificial intelligence (AI) into critical domains such as healthcare holds immense promise. Nevertheless, significant challenges must be addressed to avoid harm, promote the well-being of individuals and societies, and ensure ethically sound and socially just technology development. Innovative approaches like Embedded Ethics, which refers to integrating ethics and social science into technology development based on interdisciplinary collaboration, are emerging to address issues of bias, transparency, misrepresentation, and more. This paper aims to develop this approach further to enable future projects to effectively deploy it. Based on the practical experience of using ethics and social science methodology in interdisciplinary AI-related healthcare consortia, this paper presents several methods that have proven helpful for embedding ethical and social science analysis and inquiry. They include (1) stakeholder analyses, (2) literature reviews, (3) ethnographic approaches, (4) peer-to-peer interviews, (5) focus groups, (6) interviews with affected groups and external stakeholders, (7) bias analyses, (8) workshops, and (9) interdisciplinary results dissemination. We believe that applying Embedded Ethics offers a pathway to stimulate reflexivity, proactively anticipate social and ethical concerns, and foster interdisciplinary inquiry into such concerns at every stage of technology development. This approach can help shape responsible, inclusive, and ethically aware technology innovation in healthcare and beyond.

## Introduction

Recent developments in artificial intelligence (AI) and particularly machine learning (ML)^1^ in health promise to revolutionize healthcare research and delivery. For example, ML has been employed for diagnostics in imaging disciplines such as radiology (e.g., Wichmann et al., 2020) and dermatology (e.g., Esteva et al., 2017); in biomarker detection (e.g., Vazquez-Levin et al., 2023); care robotics (e.g., Denecke & Baudoin, 2022); and neurotechnology (e.g., Sejnowski et al., 2014). However, ethical and social challenges have arisen, such as risks of algorithmic discrimination, unfair distribution of the burden and benefits of the new technologies, privacy infringements, environmental consequences, or economic conflicts of interest, to name but a few (Broussard, 2023; D’Ignazio & Klein, 2020; Noble, 2018; O’Neil, 2016). Although the challenges and potential harms of AI in the healthcare context have been broadly discussed in the literature (European Parliamentary Research Service, 2022; McLennan et al., 2018), there, to date, are no established systemic methods to identify and tackle them effectively in the concrete contexts of technology development and deployment (McLennan et al., 2020a, 2020b).

Numerous national and international research groups, policymakers, and governmental organizations have issued frameworks for researchers and developers aimed at ensuring the ethical development of health technologies (e.g., Char et al., 2020; Jobin et al., 2019; Vayena et al., 2018) and a range of other approaches in the comparably young discipline of applied AI ethics use theoretical and empirical ethics and social science methods to mitigate the potential harms of new technologies. These include various AI guidelines (Jobin et al., 2019), impact assessment approaches (Ada Lovelace Institute, 2022; Lucivero et al., 2011), as well as integrated and more holistic frameworks like “responsible research and innovation” (von Schomberg, 2013) and socio-technical integration research (Fisher & Schuurbiers, 2013). Most recently, the notion of the “house ethicist” (Valiña et al., 2023), and more tangible approaches (Afroogh et al., 2023; Bleher & Braun, 2023; Brey & Dainow, 2023; Tigard et al., 2023), have been proposed. These works are accompanied by a range of theoretical discussions, which provide helpful reflection about the new ways of integrating ethical and social science analysis and inquiry. This includes, for example, discussions of translational ethics and medicine (Baroe, 2014; Cribb, 2010; Kagarise & Sheldon, 2000; Kremling et al., 2023); discussions on transformative ethics for better implementation of normative requirements in biomedical and technology development practice (Kuehlmeyer et al., 2023); and discussions about the co-constructive effects of algorithmic impact assessments providing meta reflection (Metcalf et al., 2021).

However, the literature still provides few “hands-on” recommendations that can be implemented. Furthermore, a frequently described drawback of analyzing ethical and social issues of technologies remains persistent: such issues can only be addressed once they have been identified; but after-the-fact analysis is often too late, as technologies and their uses have already had real-world effects, including harm (Collingridge, 1980). Hence, while post-implementation analysis certainly helps mitigate adverse effects, it is criticized for lacking forward looking strategies to effectively avoid harm before it occurs.

On a regulatory level, a lengthy discussion of ethical and social issues of AI has resulted in the recent efforts around the EU AI Act (European Parliament, 2023), a governance document to protect the European Union’s citizens from the potential adverse effects of AI technologies. It is recognized as one of the most influential steps to regulate AI to date (Floridi, 2021; Novelli et al., 2023) and will come into effect in 2025. However, in its current state, the AI Act has already been criticized for the lack of concrete strategies that can readily be implemented to address ethical and social challenges once they have been identified (Kudina, 2021; Floridi, 2022).

In light of these challenges, a more dynamic approach than the existing frameworks and regulations is needed to put ethical principles into action and help ensure that the ethical and social issues that emerge during different stages of the research and development process are addressed. To fill this gap, we have proposed the Embedded Ethics approach (Fiske et al., 2020; McLennan et al., 2022, 2020a, 2020b), and since developed it into the Embedded Ethics and Social Science approach (Breuer et al., 2023). Our approach combines ethics and social science analysis to identify and address challenges posed by AI in their specific context of development and application. In the tradition of embedded ethical, legal, and social (ELSI) research, as it was established during the human genome project (Dolan et al., 2022) and larger nanotechnology initiatives (Viseu, 2015), the term “denote[s] the practice of integrating the consideration of social and ethical into the entire development process in a deeply integrated, collaborative and interdisciplinary way” (McLennan et al., 2020a, 2020b, p. 488). The approach utilizes empirical methodological and conceptual frameworks from bioethics and the social sciences (particularly Science & Technology Studies) to help interdisciplinary consortia anticipate harmful effects and suggest new ways of thinking about ethical and social challenges during development processes.

The key element of the approach is having trained empirical ethicists and/or social scientists embedded among the project partner’s research and development processes to conduct empirical and normative analysis of ethical and social issues. By participating in research and development processes and iteratively moving between established ethical and social debates in the literature and specific development problems, the embedded researcher(s) support the development teams in recognizing and addressing ethical and social concerns as they emerge. Embedding researchers into health AI projects has proven particularly productive in encouraging sensitivity to newfound concerns, preventing ethical analysis from being an afterthought, and bringing ethical and social science thinking closer to the workbench (Tigard, 2022). To that end, interaction with AI researchers and domain specialists, such as clinicians, should be fostered from the outset.

To gain access to project teams’ structures and workflows, embedded researchers participate in regular team meetings and carry out their work in the same venue when possible. Such regular exchanges with the research team allow the embedded researchers to develop a profound understanding of the procedural and technological details of the research project and the standard practices and procedures. Additionally, active participation in the meetings creates opportunities to identify ethical and social issues that might arise during the research project and to directly clarify technical details that might affect them. Moreover, spending time in the teams' environments also helps embedded researchers to familiarize themselves with their colleagues’ working methods and to participate in their day-to-day work on a social level. It further provides opportunities for one-on-one discussions about ongoing ethical and social analyses with individual team members.

Since piloting the approach at the Technical University of Munich in 2019, we have conducted a total of seven Embedded Ethics and Social Science projects within various interdisciplinary consortia in the field of health AI projects (see Textbox 1). By applying a wide range of methods, we have learned how to incorporate the analysis of ethical and social issues into health AI projects in a dynamic, practice-oriented way. Based on these experiences, we now propose a set of methods that have, in various combinations, proven successful in embedding ethics and social science research in health AI projects. With this, we aim to offer a toolbox for embedded ethics and social science scholars to learn and select individual tools and combinations of tools that benefit their own projects.

**Textbox 1:** Embedded ethics and social science projects

**DR-AI:** The Dermatology-Radiology-AI project set out to research and develop ML-based diagnostic tools for clinical dermatology and radiology. The ethical arm of DR-AI actively engaged in the project’s strategic discussions and included empirical research at the clinic to enrich the project with qualitative insights and assess DR-AI team members’ perspectives and ethical considerations related to the project.
**INTERVENE:** The interdisciplinary research project INTERnational consortium for integratiVE geNomics prEdiction combined AI with large amounts of genomic and other health data to develop polygenic risk scores, to better prevent complex and genetic diseases, and to personalize diagnostics and treatment. The project aimed to increase the predictability and applicability of screening and the comprehensibility of genetic risk scores for citizens and clinicians. INTERVENE's ethics work package advised and supported the consortium on ethical issues within the research project. It explored the ethical and social implications of AI in genomics and polygenic risk scores for clinical and public health settings.
**NEUROTECH:** The TUM’s Innovation Network for Neurotechnology in Mental Health develops innovative approaches to treat mental dysfunction in working memory loss, depression, and pain. The project includes developing and deploying multimodal sensors, mechanistic models of neural circuits, and individualized computational treatment strategies. The "Neuro-ethics: Embedded Ethics and Social Science for Responsible Neurotechnology" work package accompanies the other project parts to monitor and examine the research and development processes of applying invasive and non-invasive neurotechnology for data collection and analysis.
**BIOMAP:** The Biomarkers for Atopic Dermatitis and Psoriasis consortium investigated the causes and molecular mechanisms of the chronic inflammatory skin diseases atopic dermatitis and psoriasis to identify relevant biomarkers. With the results, the project developed tools for optimized, targeted treatment decisions. The ethics work of BIOMAP focused on examining – theoretically and empirically – the ethical and social aspects of biomarker research and application, particularly regarding patient stratification, the use of large amounts of health data, and predictive tools in healthcare.
**Geriatronics Lighthouse Initiative:** The multi-consortial Geriatronics Lighthouse Initiative set out to develop intelligent robotics that can assist older adults in living a self-determined life. The Responsible Robotics project (RR-AI) was the embedded ethics and social science activity in this initiative. It empirically studied the ethical, social, and legal dimensions of AI-based technologies developed in the initiative, including basic research, and the practical development of telemedicine and personal robotic assistant systems (i.e., GARMI), and implementation considerations.
**METHAD:** The project Toward a MEdical ETHical ADvisory system developed an algorithm for assisting with moral decision-making in dilemmas frequently occurring in medical institutions. The research team consisted of two software engineers, an ethicist, and a medical doctor who chose the underlying moral framework, uncovered relevant variables, developed a ML model, and trained it with data from clinical cases.
**MedAIcine:** MedAIcine is the pilot project of the Center for Responsible AI Technologies. It integrates STS, ethics, philosophy, and computer science research to critically examine concepts and conflicts regarding AI’s responsible design and use in medical imaging. The project uses the embedded ethics and social science framework to research how questions of trust, bias, fairness, explainability, and vulnerability emerge in two case studies – research projects on AI in radiology (AI in Medicine Lab, TUM) and dermatology (OCTOLAB, University of Augsburg) into which members of the MedAIcine team are embedded.

## Goals per Project Stage

The Embedded Ethics and Social Science approach is dynamic and needs to be adapted to the needs and goals of each individual project. The framework promotes several goals that correspond to the typical project stages of research projects. Three main project stages are usually distinguished: 1) project design, proposal, and preparation; 2) research and development; and 3) evaluation and dissemination of results. At each stage, the approach promotes the identification, evaluation, and addressing of ethical and social issues as they emerge during the process.

### Project Design, Proposal, and Preparation

In the first stage of a project, an embedded researcher’s goal is to (1) familiarize themselves with the project, (2) map the research topic and objective as well and (3) the constellation of project stakeholders. They will further review thematically relevant ethics and social science literature to ensure they are familiar with the respective state of the art. These processes might need to be repeated once the project has been approved and initiated. Another equally important milestone in this initial stage is to define the embedded researcher’s role within the project and synchronize all project members’ expectations. To achieve this, the embedded researcher chooses and/or amends suitable methods from this toolbox, designs an appropriate project plan, and discusses it with interdisciplinary project colleagues. Through this process, differing goals can be identified, and misunderstandings can be addressed so that stakeholders gain a mutual understanding of each other’s role and practices in the project.

## Research and Development

The objectives of the second project stage are to identify ethical and social issues that arise during the research and technology development process. Often, issues arise that could not have been foreseen in the planning stage, or certain issues articulate themselves differently than expected. During this project stage, embedded researchers use a range of empirical ethics and social science methods to (1) accompany and analyze the epistemic and social processes in the project, (2) provide structured feedback to project colleagues in engineering and medicine and (3) come to agreements about how to adjust project processes to address the ethical and social issues identified in their analysis. Thus, the embedded researcher’s tasks at this stage include identifying, communicating and collaboratively addressing project-specific ethical and social issues, inspiring reflexivity in development (Stilgoe et al., 2013), and providing normative recommendations for decision-making or moral justifications for a specific course of action based on empirical insights. Applied ethics approaches, like Wide Reflective Equilibrium (Doorn & Taebi, 2018), can help weigh recommendations and justifications. At the end of this stage, the interdisciplinary team should have a comprehensive overview of the project’s ethical and social issues based on theoretical and empirical results and, together with the team, establish pathways to address critical, potentially harm-inducing components of the technology under development.

### Evaluation and Dissemination

The overall goals during the third project stage are (1) to evaluate in what way the issues revealed in the research and development stage are accounted for, (2) to ensure that limitations of the research results and/or products are thoroughly reported, (3) to stimulate wider stakeholder and public discussions on the identified ethical and social issues and (4) publish the results of the project, including challenges and limitations, jointly with engineering and medical researchers as well as separately for specialist audiences. In this final stage of the project, the embedded researcher leads the establishment of collective processes of evaluation, ensuring specifically that challenges and limitations are accounted for, organizes workshops to discuss results with diverse stakeholders and the public and leads as well as contributes to processes of disseminating the results to diverse scientific audiences, including the public, different scientific fields, and policy and regulatory bodies.

For these diverse tasks, we employ a broadly abductive approach (Tavory & Timmermans, 2014). That is, our analysis is driven by the research goals and engages with existing findings, principles and concepts in the literature, while also seeking to identify unanticipated themes and generate novel concepts applying a “bottom-up” grounded theory-based approach (Charmaz, 2006). Our analysis is iterative and highly collaborative, with novel findings informing subsequent steps of data collection and analysis and discussion and planning processes in the project. It is essential that researchers receive appropriate training in the respective methods. We do not recommend that researchers without specific methodological training in the respective empirical methods utilize these approaches, as this will lead to poor research outcomes.

## Embedded Ethics and Social Science Toolbox

Over seven projects, our research groups have applied different methods to achieve the aforementioned goals of Embedded Ethics and Social Science. In the following section, we introduce nine tools that have proven beneficial in our projects and what we have achieved by applying them. Together, these methods and associated lessons learned provide an expandable toolbox to inform future Embedded Ethics and Social Science projects (see Table 1 for an overview).

**Table 1:** Overview of the toolbox

Tool	Rationale	Service for project goals	How-to
Stakeholder analysis	To assess stakeholder constellations, interactions, and interests	Identify stakeholder-specific expectations, perspectives, and roles in the project	Analyze who is involved in, affected by, or interested in the project’s outcomes and effects
Literature review	To create systematic descriptions of previously identified ethical and social issues OR To synthesize results of existing empirical bioethics and social science research OR To identify reasons and conclusions in the argument-based literature for conceptual questions	Create a shared knowledge base among the team members	The type of literature review depends on the research questions
Ethnographic approaches	To study the practices and processes of the researchers involved in the project	Understand key points in research and development practices where ethical and social issues surface and relevant decisions are made	Participate in, observe, document, and analyze the social and epistemic practices of research
Peer-to-peer interviews	To assess project participants´ perspectives and experiences as well as factors shaping their practices	Identify explicit and implicit values as well as structural conditions that guide research and development	Qualitative, semi-structured interviews with members of the project
Focus groups	To assess the perspectives and needs of diverse publics and stakeholders	Get nuanced insights into the public’s views and experts in the field	Moderated group discussions allow for discursive dynamics to assess the views on the technology under development, possibly scenario-based
Interviews with affected groups and external stakeholders	To assess the perspectives and needs of diverse publics and stakeholders	Contextualize the normative analysis Identify gaps in the literature (in combination with a literature review) Help ensure that recommendations are relevant for policy and clinical practice	Qualitative interviews that are analyzed with a thematic analysis or grounded theory approach
Bias analysis	To identify potentially inscribed technological and societal biases in datasets	Inform research decisions that aim to reduce biases and inform future developers about the limitations of the research outputs	Check the representativeness of the data set for its intended use and user group together with developers and engineers
Workshops	To present and discuss the results obtained with empirical and normative methods To facilitate dialogue and problem-solving between project members and stakeholders	Feed findings back to the ongoing development process; use a suitable workshop format to facilitate engagement and problem-solving	Set up a longer virtual or in-person meeting with all members of the project team and possibly external stakeholders Discuss findings using suitable methods, such as model cards, data sheets, or LEGO® Serious Play®
Interdisciplinary result dissemination	To foster interdisciplinary uptake of project results	Promote collaborations between the embedded ethicist and other researchers in the project Ensure the results are also addressed to technical and domain-specific communities	Co-author papers that can be read by researchers from multiple fields and/or societal stakeholders; develop shared policy recommendations

### Literature Reviews

Literature reviews are a vital tool for Embedded Ethics and Social Science projects. The goals of literature reviews are to map and analyze the current state-of-the-art in the social science and ethics literature on topics that are of relevance for the project, including concepts, theories, case studies and methodological approaches. Literature reviews can utilize or combine many kinds of search and analytic strategies, from scoping or systematic reviews to more selective approaches, such as narrative reviews, that do not aim to be exhaustive in the same way (Mcdougall, 2014; Mertz et al., 2016; Strech & Sofaer, 2012). In our projects, these reviews have been beneficial for creating a shared knowledge base for team members, including embedded researchers, on ethical and social issues relevant to the project and for developing directions for empirical studies and normative analyses. By providing a comprehensive summary of the existing ethical and social science analysis among all active project members, the risk that relevant aspects are not considered is reduced. Further, discussing the ethical arguments and social issues retrieved in a literature review provides the opportunity to collectively interrogate the project’s goals. Literature reviews can also inform the scientific community beyond the project. For example, the literature reviews we conducted for the DR-AI and BIOMAP projects have been published in leading dermatological journals to inform the community about specific risks and benefits of dermatological AI (Willem et al., 2022), and biomarker research and application for chronic inflammatory skin diseases (Fritzsche et al., 2022). Finally, publishing the results in bioethics and social science outlets contributes to the state of the art of these fields by identifying gaps in the scholarly literature and mapping future directions.

### Stakeholder Analyses

Ethical and social challenges are complicated, often opaque, and typically arise from project particularities and the interactions between stakeholders (e.g. researchers, patients, physicians, ethics committees, companies, etc.) and their interests and commitments. Stakeholder analyses have proven useful in assessing the ethical and social implications in medical AI projects specifically and in health innovation more generally (Franco-Trigo et al., 2020), as well as in corporate environments implementing Corporate Social Responsibility strategies (Mason & Simmons, 2014). To assess the stakeholders of a project, researchers analyze who might be involved in, affected by, and/or interested in the project’s outcomes and effects. The stakeholder analysis then reveals potential dependencies between the effects scientific projects have and those affected. It is a starting point to identify converging or diverging values, interests, and commitments in a (research) project. Particularly in the early stages of a project, stakeholder analyses helped us obtain overviews of the project structures, the groups and individuals involved or affected, their interests, and their dependencies (Varvasovszky & Brugha, 2000). Based on such assessments, we have identified potential sources of friction and complex ethical and social issues relevant to our projects. After investigating these frictions via other methods, such as ethnographic fieldwork or interviews, they served as a basis for analysis, the results of which were then fed back to the other members of the research group. These feedback loops may include workshops on sensitive topics, individual ad-hoc feedback, and/or collaborative feedback provided through group discussion settings.

### Ethnographic Approaches

To gain a deeper understanding of the social and ethical effects of technology under development, a flexible and iterative methodology that combines different qualitative social science methods is necessary. Ethnographic methods such as participant observation (Bryman, 2016) have proven useful in our projects as they immerse the embedded researcher deeply into the medical AI project. For instance, during participant observation, the embedded researcher shadows and documents social situations and epistemic decision-making processes that are part of the project partners’ work routines or early testing of the ML applications in (simulated) clinical practice. Field notes are then analyzed for studying the practices and processes medical AI researchers employ to develop and apply AI technologies and ask questions about why certain approaches are chosen and certain decisions made. This helps us to shed light on the social, cultural, and organizational factors shaping research and technology development. For example, in the NEUROTECH project (Ploner et al., 2023), we employed ethnographic analysis to examine the relationships between neuroscience researchers and their patients. Our analysis allowed us to identify gaps in the current understanding of endpoints of clinical trials using brain implants and the resulting implications, such as the question of maintenance or explanation. From there, we derived the need for exit plans and identified the appropriate stakeholders who should participate in creating these plans.

### Peer-to-peer Interviews

The reflexive peer-to-peer interview method (Fochler et al., 2016; Müller & Kenney, 2014), a version of the semi-structured active interview, can be particularly useful in identifying latent values and norms that guide research and technology development practices. This conversational interview method discusses (1) the individual biographies of researchers that have led them to work with their current topic, (2) their everyday work practices and how and why they organize them in certain ways, (3) factors inside and outside academia that shape their everyday work practices, and (4) their understanding of the larger purpose, meaning and impact of their work in science and society. During the interview, the interviewer aims to open up a reflexive space in which both the interviewer and the interviewee can reflexively interrogate that which otherwise might seem self-evident, every day, or “just so,” creating a process that allows the latent and implicit structure of value and meaning to surface. Furthermore, during both the interview and the analysis, the interviewer remains cognizant of the collegial relationship between the interviewer and the interviewee, which can engender trust, but also lead to specific silences when, for example, interviewees assume certain things as given or are uncomfortable sharing certain aspects of their experiences with a colleague. Interviewers must be cognizant of such challenges and request elaboration if necessary while at the same time respecting the working relationships and what individual interviewees may or may not wish to share (Tigard et al., 2023). For example, in the Geriatronics Lighthouse Initiative, reflexive peer-to-peer interviews were instrumental for analyzing what kind of “imaginaries of healthcare,” i.e., socially and culturally shaped ways of perceiving healthcare, researchers held, and how these translated into design narratives and ultimately created certain visions of healthcare robotics and not others (Breuer et al., 2023). Subsequent interventions then focused on fostering a more nuanced and diverse understanding of healthcare practice by bringing together researchers and healthcare practitioners in workshops to discuss the challenges and opportunities of robotics and ML in healthcare in real-world contexts (see 3.8. workshops).

### Focus Groups

Beyond analyzing the concrete process of research in health AI projects, in our projects, we have employed focus groups to bring in and assess the perspectives of stakeholders who are not part of the project team. In some cases, we additionally invite external experts from the discipline we are examining (Bloor et al., 2001). Focus groups are moderated group discussions in which participants can build on and/or respond to each other’s points, stimulating their thinking and enabling insights into discursive dynamics and subject positions while discussing a topic. Embedded Ethics and Social Science projects usually revolve around topics of emerging technologies, domains in which participants often do not have prior experience. Here, a scenario-based approach is particularly well suited because it gives participants something concrete to deliberate on (Felt et al., 2014). The scenarios discussed with the focus group participants are tangible examples of the emerging technology already in place or future scenarios. These scenarios can be informed by the corresponding research project’s outputs and anticipated outcomes of an emerging technology to provide participants with a realistic idea of applications currently being developed in the laboratory (Braun et al., 2022). The stakeholders’ perspectives are then analyzed to inform the project’s integrated social, ethical, and technological research. In our project in the Geriatronics Lighthouse Initiative, focus groups of nursing students provided insights into their views as potential future users. Despite their lack of prior experience with robotics and ML technology, participants developed their ideas: They raised concerns when they were presented with concrete scenarios of technological systems being developed in the Geriatronics Lighthouse Initiative (Braun et al., 2022). They suggested, for example, different application setups the research team had not thought of but that would be instrumental from a practical nursing perspective. These focus groups demonstrated the valuable contribution nurses can make to discussions of robot design. Here, focus groups served as a basis for later workshops where we brought nurses and robotics researchers together to facilitate a dialogical user engagement mode, as suggested by Breuer et al. (2023).

### Interviews with Affected Groups and External Stakeholders

The Embedded Ethics and Social Science approach is typically applied in use-case-centered research projects, where the developed technology or intervention is intended to be applied in “real-world” situations. The normative analysis in such projects must balance various considerations, including ethically and socially relevant facts and experiences. Notably, in real-world situations – while ethicists and social scientists can play a role in highlighting important issues – the decision of whether the right balance has been reached will often be up to the stakeholders. Depending on the project, we apply thematic content analysis (Braun & Clarke, 2006) and/or grounded theory approaches (Charmaz, 2006) to conduct and analyze semi-structured qualitative interviews with stakeholders, particularly with groups affected by the technology. The analysis of interviews with affected groups and external stakeholders is a crucial tool to (a) contextualize the normative and empirical analysis by providing an in-depth and practice-orientated view and experience; (b) explore views and experiences that might not yet be reflected in the literature; and (c) help ensure that recommendations are not only scientifically robust and system relevant, but also likely to influence policy and practice. For example, in BIOMAP, building on a systematic review (Fritzsche et al., 2022), we conducted expert interviews with external advisors, patient group representatives, and other stakeholders involved in biomarker research for chronic skin diseases, including early career and senior researchers, data analysts, as well as pharmaceutical industry representatives, and members of the advisory boards. These interviews aimed to provide in-depth insights into ethical and social implications of biomarker research and application, capturing diverse perspectives. They shed light on the interconnection of ethical, translational, and scientific challenges in this context (Hangel et al., 2024). The insights of the interviews together with further research results are used to inform recommendations for policy development.

### Bias Analyses

A well-established problem of ML is inscribed bias, which usually stems from problematic datasets and can have adverse effects, for example, by categorically disadvantaging people of color or other historically marginalized groups, potentially underrepresented in the data (Noble, 2018). Therefore, conducting bias analysis is an integral part of Embedded Ethics and Social Science projects. For our bias analyses, we turn to the rich body of established bias analysis frameworks (e.g., Mitchell et al., 2019; Nazer et al., 2023; Obermeyer et al., 2021) and adapt the methods we chose to work with according to the needs of our project. This way, we check the representativeness of the data set used for the model’s intended use and user group. Note, however, that a good bias analysis not only considers metadata features specified in the data set used for training but also investigates what may be absent. When developers overlook the significance of a specific parameter, they will not include it in the interface for data input, which often means that users are later unable to convey this information to the algorithm. This could lead to unjust outcomes in clinical practice. To address this issue, we also conduct bias analyses regarding the so-called omitted-variable bias (Mehrabi et al., 2019). The goal of these analyses is the preparation of exhaustive lists of parameters encompassing any and all potential factors of influence. If in doubt, our approach aims to err on the side of caution—for the subsequent removal of a parameter that may later turn out to be insignificant is far less problematic than the failure to include a relevant one. The analytical outputs of our bias analyses are prepared as short presentations targeted at the developers of our project and discussed in joint project meetings to inform research decisions that aim to reduce biases and to inform future developers about any limitations of the research outputs. 

For example, the software developed in the METHAD project, like many other algorithms employed in medical contexts, relies on specific variables, i.e., clinical parameters relevant to the model's task. In this project, we carefully considered the ethical and social relevance of potentially omitted variables, including the relevant ones in the dataset, and input them as categories in the user interface. For example, to assess whether a treatment should be performed, it would be wrong to only consider potentially increased life expectancy. Rather, the algorithm should also include estimates about the post-treatment quality of life (Hein et al., 2022; Meier et al., 2022). As another example, in MedAIcine (Jörg et al., 2023), we have performed bias analyses in unsupervised anomaly detection (UAD) models for chest X-ray analysis (Meissen et al., 2024). Testing how subgroup composition impacts UAD model performance across protected variables (sex, race, age) and their intersections, we found compounding adverse effects in intersectional subgroups: e.g., the difference in model performance between male and female patients was larger in old patients than in young ones, resulting in old female patients being at a particular disadvantage. These findings highlight the critical need for more nuanced approaches in medical ML that address intersectional biases to ensure equitable model performance across diverse patient groups.

### Workshops

To consider results from interview studies, focus group sessions, and ethnographic research for the ongoing project and *embed* them into the ongoing development process, they need to be quickly fed back to the project team. This happens, for instance, in regular update meetings, but we have also used thematically focused workshops to discuss findings in-depth and collaboratively work out ways to address the social and ethical challenges we identified. Workshops can range in length from 2 h to whole days and can be internally or externally moderated, employing different methodological set-ups to achieve collaborative problem-solving. These engagements provide a space for project members to share insights and address social and ethical questions through transdisciplinary dialogue. For example, in the DR-AI project, we developed workshops based on the ideas of model cards (Mitchell et al., 2019) and data sheets (Gebru et al., 2021) to facilitate structured, transdisciplinary dialogues to assess information on different aspects of trained models and databases, such as their intended use, out-of-scope use, factors that might influence results obtained using the model/dataset, and ethical considerations and recommendations. This format addresses a standing difficulty in health AI development, which is the tools’ context dependency and the lack of communication about the intended uses and limitations of models and data sets. The discussions at the workshop informed the limitation section of a co-authored publication, for which a model card was created and added as an appendix (Cheslerean-Boghiu et al., 2023). Thus, future computer scientists can immediately see important parameters, such as intersectional performance, when evaluating the published model for their work. In comparison, in other projects, including the NEUROTECH network and the RR-AI project on responsible robotics, we employed LEGO® Serious Play® (De Saille et al., 2022). This method has proven particularly productive for fostering reflection on researchers’ and other stakeholders’ imaginaries of technologies under development and of their future use. For instance in the RR-AI project, the participants – primarily robotics engineers and nursing professionals – were provided with sets of LEGO® bricks and given a challenge that they had to achieve together: to build a care robot. The practical work on the LEGO® model facilitated collaboration. Afterward, each group presented their model and shared their model’s story and intent, sparking discussion about the values and goals their model embodied. To conclude the workshop, participants created a shared framework for what responsible research on robotics might mean in the future and that all participants could commit to. The framework addressed procedural aspects such as user orientation, early integration of ethical and social considerations, and care-centered design but also included values such as compassion, autonomy, and transparency. The framework served as a guide to re-orientate the ongoing work in the project.

### Interdisciplinary Results Dissemination

An integral goal of the Embedded Ethics and Social Science approach is presenting the scientific outcomes of technology projects with a dedicated focus on the ethical and social dimensions. To this end, interdisciplinary publications should be pursued. Working on such joint interdisciplinary publications promotes ongoing collaborations between the embedded researchers, the engineers, and medical scholars. It ensures that the normative insights and empirical results created in the project are also effectively communicated to technical and domain-specific audiences. In the RR-AI project on responsible robotics, for example, publications frequently resulted from interdisciplinary co-authorship and were directed towards diverse (inter)disciplinary audiences. Early programmatic papers (McLennan et al., 2020a, 2020b), authored collaboratively by researchers from medical ethics, STS, and robotics, laid the basis for subsequent collaborations among the coauthors. Analyses of interviews and ethnographic observations of engineers from the Geriatronics Lighthouse Initiative Initiative were written by the social scientists and ethicists of the RR-AI project but published in an interdisciplinary journal with the goal of making analytical concepts and findings from the social sciences and ethics accessible to the human–computer interaction community (Breuer et al., 2023). Crucially, interdisciplinary collaboration extended to joint research efforts integrating stakeholder perspectives, legal analysis, and technological development, which resulted in the publication of design requirements for a data recorder for a service humanoid robot assisting in healthcare (Skerlj et al., 2023). Additional interdisciplinary publications involved collective meta-reflections on the collaboration itself, offering insights into best practices for future Embedded Ethics and Social Science work (Breuer et al., 2024; Tigard et al., 2023).

## Limitations and Strengths of Embedded Ethics and Social Science

The Embedded Ethics and Social Science approach offers a systemic, dynamic approach and hands-on recommendations for identifying and addressing ethical and social issues emerging in health AI research. It offers an integrated approach to studying technology in development that benefits significantly from combining expertise from medical ethics, bioethics, and social science fields such as STS. For this approach to be effective, a robust methodological set-up is needed. The tools we have presented in this paper each have proven useful for our identifying, discussing, calibrating, and counteracting social and ethical issues in AI healthcare projects and, thus, constitute a tested methodological toolbox. However, they are by no means the entirety of what we understand this approach to be or become. Since the submission of this manuscript, we have, for example, been exploring additional tools like responsible AI-inspired "maturity models," ethical impact assessments, and evolving the role of the embedded ethicist to a project internal “ethics reference person,” who moderates detailed discussions on project-relevant ethics. We hope that as new science emerges about integrating social and ethical considerations into ongoing projects, new, as well as existing tools will be added to this toolbox.

Even though we highlight how we interpreted some of their features and how their application was particularly productive to the Embedded Ethics and Social Science approach, the tools we present are not exclusive to this approach. The distinctive feature of the approach is that it aims to provide practical normative guidance for all project phases based on empirical inquiry. By utilizing this approach, we move away from evaluating snapshots of technological development processes in isolation; instead, our approach seeks to take on a holistic view of the project, related existing ethics and social science research, the research object, its stakeholders, and potentially affected people. By iteratively moving from empirical phases of projects to normative recommendations, embedded researchers carefully craft arguments for critical decisions that guide the research and development team toward what might be ethically desirable and socially responsible solutions.

Considering this broad range of tasks, it is essential to delineate embedded researchers’ responsibility. In our experiences, colleagues from technical fields often wish embedded researchers could substitute for what they believe to be external ethics advisory board’s tasks, e.g., judging their research ethics. Research ethical aspects are pertinent and contribute to developing emerging technologies in ways that promote the overall ethicality of the projects and, therefore, cannot wholly be disregarded by embedded researchers. However, to be able to conduct focused research, it is vital to establish a clear project plan and demarcate the embedded researcher´s range of tasks (see Project Design, Proposal, and Preparation). While these tasks can include offering ad hoc ethics consulting or other more general service-like ethics work for the consortium, we understand in-depth analysis and discussion of potential adverse social and ethical effects related to the consortium’s shared object of research as central to the approach. The range of tasks embedded researchers can take on largely depends on the time capacity they bring to the project. Depending on the project's scope, we recommend at least one full-time position for the whole project duration. However, we recognize that not all projects can allocate substantial resources to ethical and social science inquiry, and we acknowledge this as a significant limitation of our approach, particularly for research projects with limited resources in general and those in low- and middle-income countries specifically. While full embeddedness will result in more thorough results and is the core idea of the approach, we advise projects with fewer resources to select specific phases of the project to embed into and to limit the analysis of observations thematically. To sustainably increase the applicability of our approach, we call on funders to appreciate the benefits of embeddedness and the appropriate resources requested in grant applications.

No matter how many resources are available, we find it important for Embedded Ethics and Social Science projects to follow several broad guidelines that can serve as best practices (Tigard et al., 2023). First, due to the interdisciplinary collaborations at the heart of the approach, there is often a risk of ineffective communication hindering otherwise meaningful exchanges. For this reason, we see the need to establish a shared working understanding of key terms in early project phases. Likewise, it will be helpful – and often necessary – for ethicists and social scientists to obtain at least a basic proficiency in the relevant technical and medical domains (McLennan et al., 2022). Second, because a robust deployment of the Embedded Ethics and Social Science approach entails highly integrated work practices and regular exchanges across disciplines, effective working modalities will necessitate a certain level of cultural adaptation from all members of the projects. For example, time management emerges as a recurrent concern in the context of technological development. Embedded researchers need to be attuned to the practical and time-related needs of their technical collaborators and embrace dynamic planning to facilitate opportune intervals of reflection. Third, efforts should be made to foster an inclusive environment, wherein all team members – whether technical, medical, ethical, or social scientific – have opportunities to actively participate in discussing and critically analyzing the social and ethical features of technologies under development. These kinds of inclusive practices help to build skills for critical reflection on ethical and social issues in development teams while also providing continual support and expertise from those more versed in such practices. Fourth, it is also important to balance the typically concrete needs of team members with more technical backgrounds with the often more abstract research interests of embedded researchers. As such, it is important to make clear the direct relevance of the ethically and socially focused discussions and workshops to maintain engagement and interest – for example, by developing highly concrete examples – but at the same time, allow a space for more theoretical ideas to emerge. Fifth and finally, as suggested above, because technological development very often takes place within an atmosphere of commerce and competition, some project partners may feel a need to protect intellectual property and emerging trade secrets. The effectiveness of Embedded Ethics and Social Science will often hinge upon open dialogue across disciplines and project members, and in this way, it is of the utmost importance to ensure that confidentiality is upheld, thereby protecting technical breakthroughs and simultaneously promoting the open exchanges that give rise to meaningful ethical and social analyses.

## Conclusion

AI promises benefits in healthcare. However, challenges need to be addressed. New processes for addressing such challenges are fortunately arising – like the Embedded Ethics and Social Science approach. In this paper, we have outlined and described several tools for implementing this approach; namely, stakeholder analyses; literature reviews; ethnographic approaches; peer-to-peer interviews; focus groups; interviews with affected groups and external stakeholders; bias analyses; workshops; and interdisciplinary result dissemination. While we believe these methods are fundamental for embedded research in medical AI and beyond, we recognize that, given the nascent stage of interdisciplinary technology development, additional tools – and future adjustments to existing tools – will be crucial for fully integrating ethical and social science analysis into the development of emerging technologies. New research on detail-oriented approaches, such as algorithmic impact assessment, promises to add further nuance to currently available tools. In addition, we hope that the Embedded Ethics and Social Science approach will continue to diversify and take root in different contexts of technology development and application. Finally, we advocate for reflecting on the implications of embedding ethical and social analysis into medical AI research itself. What does it mean to work in an embedded way, what opportunities arise, and which limitations? Existing disciplinary problems, such as the Collingridge dilemma addressed in this paper, need practice-oriented solutions. We show how Embedded Ethics and Social Science can provide a way to stimulate reflexivity, anticipate social and ethical issues, and ultimately foster a new sensitivity to ethical and social challenges at all stages of AI development in healthcare and beyond.

## Footnotes

1 Please note that throughout this manuscript, we use both AI and ML to refer to the subject of research in the projects we use as our empirical examples. We are aware that ML is only a fragment of AI, and most projects we will touch upon are considered ML projects. Additionally, we are aware of an increasingly strong pushback against the AI term that is currently entering the public debate (e.g., https://www.newyorker.com/science/annals-of-artificial-intelligence/there-is-no-ai). However, in some instances, the term ML would be too narrow for our purposes, so in these instances, we opted for the broader AI term. We hope our readers view this decision as an opening for conversation.
